# Development of a CORe outcome set for clinical trials of RECTal cancer treatment: protocol for the CORRECT initiative

**DOI:** 10.1136/bmjopen-2025-103072

**Published:** 2026-04-13

**Authors:** Richard Garfinkle, Manju George, Krishan Jethwa, Peter Johansen, Mary Lakaszawski, Arun Nagarajan, Neil Smart, Patricia Sylla, Te Vuong, Marylise Boutros, Dean A Fergusson

**Affiliations:** 1 Division of Colon and Rectal Surgery, Jewish General Hospital, Montreal, Québec, Canada; 2 Paltown Development Foundation/COLONTOWN, Crownsville, Maryland, USA; 3 Department of Radiation Oncology, Mayo Clinic, Rochester, Minnesota, USA; 4 Department of Anthropology, McGill University, Montreal, Québec, Canada; 5 Department of Surgery, Mount Sinai Hospital, New York City, New York, USA; 6 Department of Haematology-Oncology, Cleveland Clinic Florida, Weston, Florida, USA; 7 Department of Colorectal Surgery, Royal Devon and Exeter NHS Foundation Trust, Exeter, UK; 8 Division of Colon and Rectal Surgery, Mount Sinai Hospital, New York City, New York, USA; 9 Department of Radiation Oncology, Jewish General Hospital, Montreal, Québec, Canada; 10 Department of Colorectal Surgery, Cleveland Clinic Florida, Weston, Florida, USA; 11 Clinical Epidemiology, Ottawa Hospital Research Institute, Ottawa, Ontario, Canada; 12 Department of Medicine, University of Ottawa, Ottawa, Ontario, Canada

**Keywords:** Colorectal surgery, Adult oncology, Delphi Technique

## Abstract

**Abstract:**

**Introduction:**

With the rapidly changing landscape of rectal cancer treatment, it is becoming increasingly challenging for clinicians to interpret and synthesise the vast amount of high-quality evidence being generated. A core outcome set (COS) for clinical trials in rectal cancer would help address issues surrounding outcome selection and reporting. The purpose of this research project is to develop a COS to be used in research comparing different treatment paradigms in the management of rectal cancer.

**Methods and analysis:**

This will be a mixed-methods project, including a systematic review, semi-structured interviews and a Delphi consensus process. The project was designed in accordance with the COMET (Core Outcome Measures in Effectiveness Trials) Handbook, which provides a framework for COS development based on existing evidence. A multidisciplinary Study Advisory Group, composed of rectal cancer providers, methodologists and patients, will oversee the project. A systematic review will be performed to identify an inclusive list of outcomes reported by researchers in previous rectal cancer trials. Outcomes will be collapsed into various core areas and domains according to the OMERACT Filter V.2.0. Semi-structured interviews with rectal cancer survivors and their partners/caregivers will help identify additional patient-centric outcomes not captured in the systematic review. Finally, after a final list of outcomes is generated, patients and healthcare professionals will be invited to participate in a Delphi process to develop the final COS.

**Ethics and dissemination:**

The study has received full approval with the Research Ethics Committee at the Integrated Health and Social Services Network for West-Central Montreal (health network responsible for the Jewish General Hospital) (REC: 2025-4377) and the Institutional Review Board of the Mount Sinai School of Medicine (IRB: STUDY-25-00515). The results of this study will be presented at national and international meetings and a manuscript will be submitted for publication in a high-impact surgery and/or oncology peer-reviewed journal.

**Trial registration number:**

The study was registered in the COMET database in December 2023 (https://www.comet-initiative.org/Studies/Details/2941). The full systematic review protocol, along with the search strategy and inclusion/exclusion criteria, was registered online in September 2023 (researchregistry.com; reviewregistry1705).

**STRENGTHS AND LIMITATIONS OF THE STUDY**

- This project will include a systematic review of previous rectal cancer trials to better understand patterns in outcome reporting.
- The Delphi process will include a diverse and multidisciplinary panel of healthcare professionals across various continents.
- Patient engagement and involvement will be a key aspect of this project, from inception to completion.
- Despite efforts to obtain a diverse patient panel for the Delphi process, patients with low socioeconomic and health literacy status and/or poor Internet access may be under-represented.
- This project will identify the core outcome measures to be studied in future rectal cancer trials; however, this study will not describe *how* these outcomes should be measured (eg, which patient-reported outcome measures to use).

## Introduction

 Rectal cancer is one of the most common malignancies in North America and comprises one-third of all incident cases of colorectal cancer.^1^ For several decades, rectal cancer has been treated with multimodal therapy including surgery, radiation and chemotherapy, which has resulted in drastically improved locoregional recurrence-free and overall survival rates.^2^,^5^ However, rectal surgery is still invasive, and the long-term functional sequelae of pelvic surgery and radiation have proven to be important determinants of health-related quality of life.^6 7^ This has led rectal cancer providers on a quest to determine the optimal management strategy for their patients, balancing the oncologic benefits and functional consequences of each treatment.

The paradigm of rectal cancer management further evolved with the introduction of non-operative management (NOM), also known as “watch-and-wait”, in the mid 2000s.^8^ Patients who achieved a pathologic complete response following neoadjuvant treatment experienced sustained disease-free survival, leading many to question whether surgery could be avoided in selected cases. Changes in the sequencing of therapy (eg, moving chemotherapy to the neoadjuvant setting, either before or after radiation) further improved tumour downstaging, allowing for organ preservation in up to 50% of patients.^9^,^11^ In select cases, residual disease after neoadjuvant treatment can also be excised using less invasive transanal techniques, sparing patients from a full proctectomy.^12 13^

With the rapidly changing landscape of rectal cancer treatment, it is becoming increasingly challenging for clinicians to interpret and synthesise the vast amount of high-quality evidence being generated. Not only are the interventions numerous, but the outcomes used are often heterogeneous and vary according to each study’s objectives, with some studies omitting important patient-centric endpoints.^14^ A core outcome set (COS) for clinical trials in rectal cancer would help address issues surrounding outcome selection and reporting. As defined by the international COMET (Core Outcome Measures in Effectiveness Trials) initiative, a COS is a minimum set of agreed-upon standardised outcomes that should be measured and reported in all trials for a specific condition.^15^ In addition, COS are suitable for use in routine care, clinical audits and research beyond randomised trials. Evidence demonstrates that COS lead to higher-quality randomised controlled trials (RCTs) by encouraging investigators to include all relevant outcomes established using consensus-based methodology.^16^ They also mitigate reporting bias, as outcomes that are not statistically significant are less likely to be fully reported in final publications.^16^ Altogether, COS allows for the capture of clinically meaningful and patient-centred outcomes and facilitates the proper synthesis of evidence to best inform treatment decisions.

Currently, a rectal cancer-specific COS does not exist. The CORMAC (Core Outcome Research Measures in Anal Cancer) study developed a COS for trials of chemoradiotherapy for anal cancer,^17^ and other investigator groups have developed COS for colorectal cancer research over 10 years ago.^18 19^ Given the nuances and specificities of rectal cancer management, none of these COS would be appropriate nor sufficient for the guidance of modern rectal cancer research. The purpose of this research project is to develop a COS to be used in research comparing different treatment paradigms in the management of rectal cancer.

## Methods and analysis

### Study design

**Figure 1:**
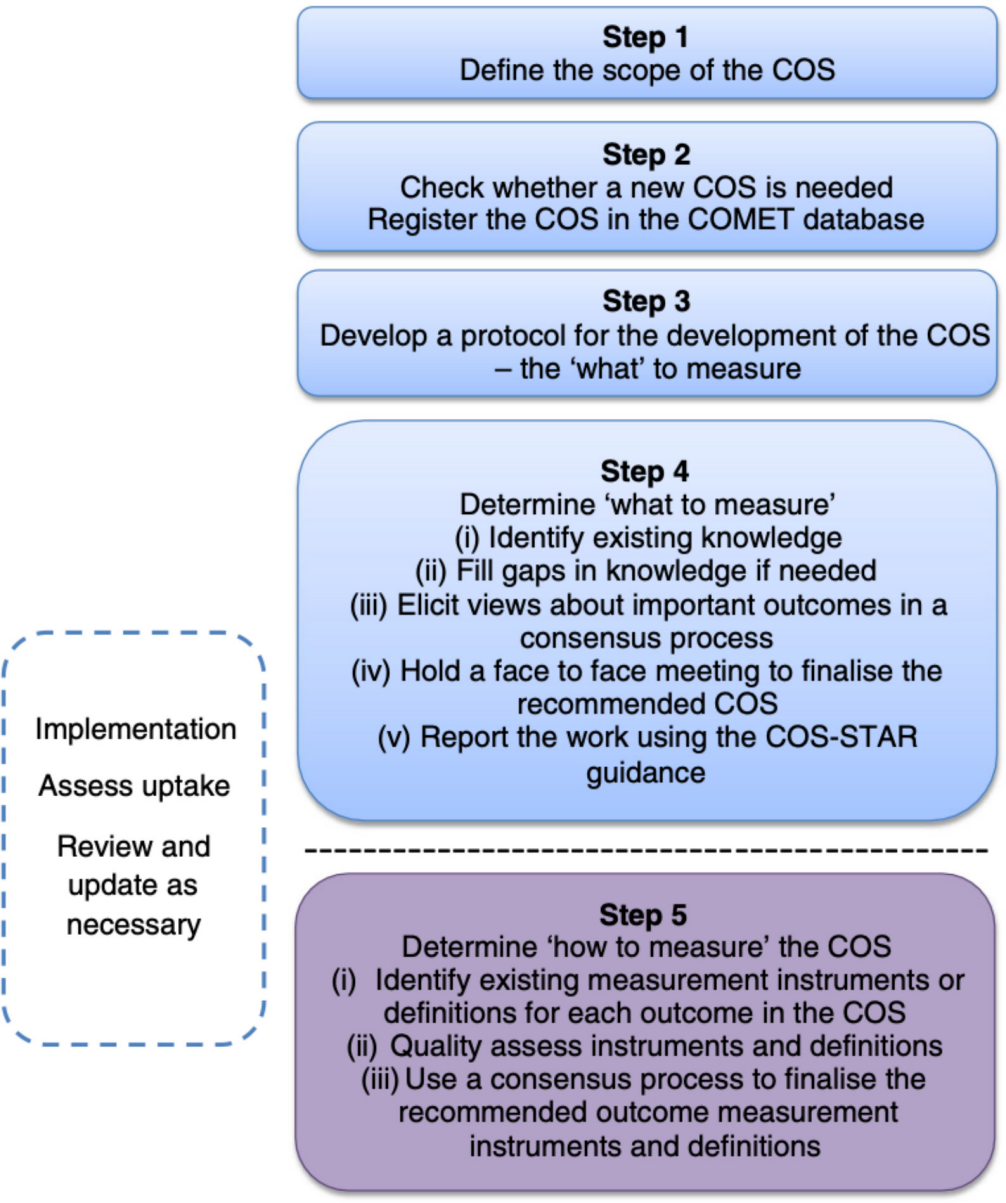
Core outcome set development process per the COMET Handbook, reused without additional changes from “Williamson *et al* Trials. 2017;18(Suppl3):820”. This article was distributed under the terms of the Creative Commons Attribution 4.0 International Licence (http://creativecommons.org/licenses/by/4.0/). COS, core outcome set; COMET, Core Outcome Measures in Effectiveness Trials.

This will be a mixed-methods project, including a systematic review, semi-structured interviews and a Delphi consensus process. The project was designed in accordance with the COMET Handbook, which provides a framework for COS development based on existing evidence.^15^ The COMET Initiative recommends a four-step process, as follows (figure 1):

#### Step 1: define the scope of the COS

The proposed COS will be developed for use in future prospective RCTs comparing different multimodal treatment paradigms in the management of adult patients with rectal adenocarcinoma. Rectal cancer is extremely rare in the paediatric population. Furthermore, other subtypes of rectal tumours are not typically treated with multimodal therapy and thus are less relevant to this COS.^20^ While COS are typically designed for use in prospective trials, the results of this project may also be applied to observational research and registries.

#### Step 2: check whether a new COS is needed and register in COMET database

A review of both PubMed and the COMET database (an online repository of completed and/or ongoing COS studies) through December 2024 did not reveal any existing COS studies specific to rectal cancer. A 2006 systematic review of trials evaluating neoadjuvant radiation therapy in rectal cancer was registered in the COMET database and ultimately published; however, this study only reported and analysed different clinical endpoints within the trials (ie, did not develop a COS), and the included studies were from 1993 to 2005.^21^ The current study protocol was subsequently registered in the COMET database in December 2023 (https://www.comet-initiative.org/Studies/Details/2941).

#### Step 3: develop a protocol for the development of the COS

The remainder of this manuscript will outline the protocol for the project. As recommended by the COMET Handbook, this will include (1) Identifying existing knowledge through the conduct of a systematic review, which for the purposes of this project, will comprise all previous outcomes reported in rectal cancer trials, (2) Filling any gaps in knowledge through qualitative research, which will comprise semi-structured interviews to ensure no relevant outcomes are missing, (3) Eliciting views of important outcomes in a consensus procedure, via Delphi process and (4) Having an in-person meeting to finalise the COS.

#### Step 4: determine “what” to measure

This will be the fully developed COS and will be reported in a subsequent manuscript and available online.

### Study advisory group

Each step of the project will be overseen by a study advisory group (SAG), which has been formed. Members of the SAG (all co-authors) reflect the multidisciplinary nature of rectal cancer care and include Colorectal Surgeons, Medical Oncologists and Radiation Oncologists with expertise in rectal cancer clinical trials. The SAG also includes an experienced Enterostomal Therapy nurse, an epidemiologist with significant experience in systematic reviews and Delphi consensus methodology and two rectal cancer patient advocates who have completed treatment.

#### Existing knowledge – systematic review

A systematic review will be performed to identify an inclusive list of outcomes reported by researchers in previous rectal cancer trials. The systematic review protocol is registered online (researchregistry.com; reviewregistry1705) and the search strategy is available for review (online supplemental file 1). Eligible studies need to meet all the following criteria: (1) RCT study design (phase I, II or III), (2) Included adult (≥18 years old) patients, (3) Diagnosed with rectal adenocarcinoma, staged clinically with pelvic MRI, (4) Who had non-metastatic and technically resectable disease and (5) The RCT was evaluating any one (or more) locoregional or systemic therapy for rectal cancer as its primary exposure. For the purposes of this review, locoregional therapies included surgery and radiation therapy, while systemic therapies included chemotherapy, immunotherapy and/or additional targeted therapies. Only RCTs published in the year 2000 and onwards will be included to best reflect contemporary rectal cancer management. Two independent reviewers will extract all outcomes verbatim from the individual studies and group similar outcomes under one name. Subsequently, outcomes will be collapsed into various core areas and domains according to the OMERACT Filter V.2.0, a conceptual framework that encompasses all that is measurable in a trial.^22^ Briefly, OMERACT Filter V.2.0 comprises three core areas (death, life impact and pathophysiological manifestations) and one recommended area (resource use). These core areas can then be further subcategorised into multiple domains. A final list of outcomes within their individual core areas and domains will be generated and will be reviewed and approved by the SAG. As secondary objectives, the frequency of each outcome will be reported, and different trends in outcome reporting will be analysed according to individual study and investigator characteristics. Further details for the review protocol can be found in the online registered protocol. The systematic review began in 2024.

#### Knowledge gaps – qualitative research

The output of the systematic review will reflect the outcomes deemed important by previous researchers; however, it is likely that relevant outcomes will be missing from this first list, especially patient-centred outcomes. It is essential to gather input from other key stakeholders in rectal cancer management, particularly patients. As such, two additional groups will be queried for relevant outcomes: (1) Rectal cancer survivors and their family members, using appropriate qualitative research methods as outlined below and (2) The SAG, who will give final approval of the outcome list.

##### Participants

Patients will be recruited from COLONTOWN, an online community and support group for patients with colorectal cancer. The executive committee of COLONTOWN gave approval for online recruitment of patients through their platform. Included patients will have completed their proposed treatment and should not be receiving any active therapy for rectal cancer. We will aim for mixed representation, including equal proportions of male and female, as well as younger and older (<60 or ≥60 years old), participants. All patients need to have undergone neoadjuvant treatment and/or a proctectomy. Patients managed with NOM will be included; however, patients who underwent local excision alone as the sole treatment for their rectal cancer will not be included. Participants will also be asked to invite their family members and/or caregivers, who often provide key insight into the lived experiences of patients.

##### Interview format

Semi-structured interviews will take place virtually (June–November 2025) using videoconference and will follow a prepared interview guide. Open-ended questions will be asked regarding the patient’s experience, both positive and negative, during their rectal cancer treatment. Patients will be encouraged to share those outcomes which mattered to them most during treatment, and what adverse effects or undesirable consequences were most impactful. The semi-structured interviews will be audio-recorded and transcribed by a member of the research team with appropriate informed consent.

##### Sample size

A convenient sample of 8–10 patient participants will be chosen for the semi-structured interviews. This number was based on the investigator’s previous experience with a focus group study on bowel dysfunction after rectal cancer surgery, which produced rich and saturated data for qualitative thematic analysis.^23^

##### Data analysis

The grounded theory approach will be used as a general framework for analysis, and the constant comparative method will be applied on review of the semi-structured interviews transcripts.^24 25^ Grounded theory is an inductive approach to theory development grounded in original data and observations that makes use of an iterative process to achieve thematic saturation. Two individual reviewers will code data based on emerging patterns, concepts and themes, which will subsequently be analysed and categorised into outcomes and domains.

##### Final outcome list

The list of outcomes generated from the qualitative interviews will be amalgamated to the outcome list generated from the systematic review and will be reviewed by the SAG in a virtual meeting. Duplications and redundancies will be removed. The SAG will have an opportunity to include any additional relevant outcomes that were not yet identified. The final outcome list will be approved by the SAG prior to dissemination in the Delphi process.

### Consensus – Delphi process

The Delphi process was selected to generate the COS and gather input on outcomes deemed important by all key stakeholders in the management of rectal cancer. The Delphi process is an iterative process where individual items can be scored or ranked according to their level of importance. In subsequent rounds, participants are privy to summary statistics pertaining to each individual item which may impact their future scores. However, unlike other methodologies, the process is anonymous and can be completed online, which eliminates the potential for dominant parties to influence the decision of others and overcomes geographic constraints. The results of the Delphi process will be reported in accordance with the newly developed Accurate Consensus Reporting Document reporting guideline for consensus-based research.^26^

#### Participants

Participants will be chosen from two key stakeholder groups: patients and healthcare professionals. In both groups, implied consent for the Delphi process will be obtained on opening the online questionnaire.

Healthcare professionals will be recruited using a convenient and snowball sampling approach. The SAG includes members with active participation from major societies, including the American and Canadian Societies of Colon and Rectal Surgeons, the American Society for Radiation Oncology, the American Society of Clinical Oncology and the Wound, Ostomy and Continence Nurses Society. Each SAG member will recruit important thought-leaders and experts in their respective fields to participate in the Delphi process. Each participant will subsequently identify two additional participants from another medical speciality at their institution who treats rectal cancer (Colorectal Surgery, Radiation Oncology, Medical Oncology, Enterostomal Therapy nurse, Colorectal Cancer pivot nurse). This will allow for a diverse panel of healthcare professionals without undue weight from any one speciality.

Patient recruitment will be through COLONTOWN (similar to the semi-structured interviews). A diverse panel of patient participants will be included according to pre-specified demographic and treatment factors (table 1). All patients need to have completed treatment for their rectal cancer. Patient participation will be completely voluntary and no identifying information will be stored beyond completion of the final Delphi round.

**Table 1:** Desired characteristics of patient participants in the Delphi process

Characteristics	Proportion of total sample (%)
Continent	
Within North America	60–70
Outside of North America	30–40
Age	
<60 years old	20–25
>60 years old	75–80
Gender	
Male	50
Female	50
Neoadjuvant therapy	
Yes	90–100
No	0–10
Managed with non-operative intent	
Yes (even if ultimately underwent surgery)	25–30
No	70–75
Status of ileostomy/colostomy	
Previous	60–80
Current	10–20
Never	10–20

#### Recruitment and sample size

Given that there are no recommendations regarding the number of Delphi participants, the sample size will be pragmatic; we will aim for 25 patient participants and 75 healthcare professionals. If additional healthcare professionals participate, we will recruit more patients to ensure no less than a 1:3 patient to healthcare professional ratio.

#### Delphi rounds

The final list of outcomes generated from the systematic review and qualitative research will be uploaded into an online questionnaire using a secure, web-based software and administered to participants virtually. The key steps and methods employed will be described to participants in the first Delphi round. Additionally, participants will answer background questions regarding demographic and disease/practice characteristics. Participants will then be asked to rate the importance of each outcome using a 9-point Likert scale, grouped into three main categories: 1–3 (limited importance), 4–6 (moderate importance, but not critical) and 7–9 (critical importance). This rating scale is recommended by the GRADE (Grading of Recommendations Assessment, Development, and Evaluation)working group and has been used in several previous COS studies. To facilitate patient participation, each outcome will be described in medical, as well as layman’s, terminology. The outcomes in the questionnaire will also be grouped according to their individual domains, and more general outcomes will precede more specific ones. In this first round only, participants will also be able to write in additional outcomes that they feel are missing and warrant a rating of “critical importance”.

The SAG will review the results of Round 1 and will decide which outcomes to retain (based on predefined criteria outlined below) or add for Round 2. For the second Delphi round, participants will be presented with summary statistics for each outcome and will be able to vote anew on each item in the Delphi. Participants will be given 2 weeks to complete each Delphi round, and 4 weeks in between each round. Reminder emails will be sent out periodically for non-responders. The Delphi will be terminated after two rounds. The Delphi process is anticipated to begin in Spring 2026.

#### Consensus

A threshold of 70% will be used for consensus when considering any outcome.^27^ After Round 1, outcomes that are rated as “limited importance” by ≥70% of participants AND rated as “critical importance” by <15% of participants will be dropped from the second round. All other outcomes will be retained for Round 2. At the conclusion of the second Delphi round, outcomes that are rated as “critical importance” by ≥70% of participants will be considered for the final COS. Outcomes meeting the 70% threshold, but which ≥15% of participants also rated as “limited importance”, will require more in-depth discussion and decision-making among the SAG.

### SAG in-person meeting for final COS

The COMET Initiative recommends one in-person meeting to review the final COS.^15^ The SAG will invite interested Delphi participants from both stakeholder groups to join the in-person meeting, where summary data will be presented from Round 2 of the Delphi. Outcomes reaching consensus for “critical importance” will be included in the final COS; however, if the outcome was also rated as “limited importance” by ≥15% of participants, it will be discussed during this meeting. Additional outcomes may be discussed by the SAG but can only be added to the final COS if 90% of SAG members agree.

### Patient and public involvement

The CORRECT initiative is committed to involving patients at every aspect of the project. Two patients are members of the SAG and are co-responsible for the design and oversight of the work. As part of the knowledge synthesis plan, patients will also participate in semi-structured interviews to elucidate additional patient-centric outcomes not otherwise identified in the systematic review. Finally, a larger group of patients will be invited to participate in the Delphi consensus process to develop the final COS. We plan on sharing our results with various rectal cancer patient advocacy groups to help disseminate our findings and encourage similar patient-centric work in other disease groups.

## Ethics and dissemination

The qualitative research (semi-structured interviews) received full approval from the Mount Sinai School of Medicine Institutional Review Board (IRB: STUDY-25-00515), and the Delphi process has received full approval with the Research Ethics Committee at the Integrated Health and Social Services Network for West-Central Montreal (health network responsible for the Jewish General Hospital) (REC: 2025-4377). Consent forms for both healthcare professionals and patient participants will be embedded into the online Delphi questionnaire, with appropriate contact information for the lead study investigators. The systematic review was deemed exempt from ethics approval.

The results of this study will be presented at national and international meetings and a manuscript will be submitted for publication in a high-impact surgery and/or oncology peer-reviewed journal.

## Supplementary material

10.1136/bmjopen-2025-103072 online supplemental file 1

## Data Availability

Data are available upon reasonable request.
