# Supplementary material for: Development of a CORe outcome set for clinical trials of RECTal cancer treatment: protocol for the CORRECT initiative
Source: BMJ Open. 2026 Apr 13;16(4):e103072. doi: 10.1136/bmjopen-2025-103072 (PMC13084911; doi:10.1136/bmjopen-2025-103072)
Supplement: online supplemental file 1 [file bmjopen-16-4-s001.docx]

[Ovid MEDLINE(R) ALL <1946 to January 03, 2024>](https://login.proxy.bib.uottawa.ca/login?url=http://ovidsp.ovid.com/ovidweb.cgi?T=JS&NEWS=N&PAGE=main&SHAREDSEARCHID=1qG5ilquWAmP2ALYA69IYzukl8gjqIOLNfdqVYRcTg4Z6yJPR7Zdyzo2SLE2Pqs6e)

1 exp Rectal Neoplasms/ 55056

2 ((rectal or rectum or anal or anus) adj2 (neoplasm* or tumo?r* or cancer or malignan* or carcinoma* or adenocarcinoma*)).tw,kf. 44989

3 1 or 2 68394

4 exp Antineoplastic Agents/ 1259994

5 exp radiotherapy/ 209519

6 exp Proctectomy/ 5376

7 exp chemoradiotherapy/ or exp chemotherapy, adjuvant/ or neoadjuvant therapy/ or radiotherapy, adjuvant/ 97307

8 (oxaliplatin or 5-FU or leucovorin or capecitabine).mp. 48708

9 5-fluorouracil.mp. 35792

10 capecitabine.mp. 9019

11 (chemotherap* or radiotherap* or radiation therap* or irradiation or chemorad* or brachytherap*).tw,kf. 926490

12 bevacizumab.mp. 23425

13 (ziv aflibercept or ramucirumab or cetuximab or panitumumab).tw,kf. 10084

14 Irinotecan.mp. 13147

15 Cetuximab.mp. 8892

16 Panitumumab.mp. 2182

17 Regorafenib.mp. 1984

18 (Trifluridine or tipiracil).mp. 998

19 exp Transanal Endoscopic Surgery/ 1066

20 (Proctectom* or surg* or resect* or excision*).tw,kf. 2669195

21 pembrolizumab.mp. 9585

22 nivolumab.mp. 10209

23 ((adjuvant or neoadjuvant) adj2 (therap* or treat*)).tw,kf. 84183

24 or/4-23 4439168

25 3 and 24 43383

26 exp Rectal Neoplasms/dt, su, rt, th 34395

27 25 or 26 48770

28 randomized controlled trial.pt. 605916

29 (randomly or randomi* or placebo*).tw. or trial.ti. 1327481

30 28 or 29 1438049

31 exp animals/ not humans/ 5182830

32 30 not 31 1313705

33 27 and 32 4004

34 limit 33 to yr="2000 -Current" 3211

[Embase Classic+Embase <1947 to 2024 January 03>](https://login.proxy.bib.uottawa.ca/login?url=http://ovidsp.ovid.com/ovidweb.cgi?T=JS&NEWS=N&PAGE=main&SHAREDSEARCHID=34cQxYCCLGXaDhqRhSGT37b44R8EfGiBpeHLroLbPSxXPsrcoIbu05nsKyooQwEzf)

1 rectum cancer/ or rectal carcinoid/ or rectal lymphoma/ or exp rectum carcinoma/ 60515

2 ((rectal or rectum or anal or anus) adj2 (neoplasm* or tumo?r* or cancer or malignan* or carcinoma* or adenocarcinoma*)).tw. 66375

3 1 or 2 84932

4 exp antineoplastic agent/ 3091088

5 exp rectum resection/ 25031

6 radiotherapy/ 207921

7 exp beam therapy/ 158489

8 exp cancer adjuvant therapy/ 154157

9 (oxaliplatin or 5-FU or leucovorin or capecitabine).tw. 67623

10 5-fluorouracil.tw. 46260

11 capecitabine.tw. 15865

12 (chemotherap* or radiotherap* or radiation therap* or irradiation or chemorad* or brachytherap*).tw. 1407571

13 bevacizumab.tw. 37151

14 (ziv aflibercept or ramucirumab or cetuximab or panitumumab).tw. 19487

15 Irinotecan.tw. 18714

16 Cetuximab.tw. 15389

17 Panitumumab.tw. 3795

18 Regorafenib.tw. 3722

19 (Trifluridine or tipiracil).tw. 1162

20 exp transanal endoscopic surgery/ 1548

21 (proctectom* or surg* or resect* or excision*).tw. 3717019

22 pembrolizumab.tw. 19297

23 nivolumab.tw. 20221

24 ((adjuvant or neoadjuvant) adj2 (therap* or treat*)).tw. 136136

25 or/4-24 7166221

26 3 and 25 63681

27 exp rectum cancer/dt, rt, su, th 24361

28 26 or 27 65177

29 (randomi* or randomly).tw. or placebo*.mp. or double-blind*.tw. or trial.ti. 2056158

30 (exp animal/ or nonhuman/) not exp human/ 8019686

31 29 not 30 1846243

32 28 and 31 6133

33 conference abstract.pt. 5018954

34 32 not 33 4276

35 limit 34 to yr="2000 -Current" 3710

[EBM Reviews - Cochrane Central Register of Controlled Trials <December 2023>](https://login.proxy.bib.uottawa.ca/login?url=http://ovidsp.ovid.com/ovidweb.cgi?T=JS&NEWS=N&PAGE=main&SHAREDSEARCHID=35dlQkHbks1hbdES0wBaiIvZY5j8oR8Ws1gwrWWWbX5L5Bf7RGiYz2gjGNiTX8Yuc)

1 exp Rectal Neoplasms/ 2487

2 ((rectal or rectum or anal or anus) adj2 (neoplasm* or tumo?r* or cancer or malignan* or carcinoma* or adenocarcinoma*)).tw,kw. 5054

3 1 or 2 5783

4 exp Antineoplastic Agents/ 78611

5 exp radiotherapy/ 10304

6 exp Proctectomy/ 206

7 exp chemoradiotherapy/ or exp chemotherapy, adjuvant/ or neoadjuvant therapy/ or radiotherapy, adjuvant/ 9382

8 (oxaliplatin or 5-FU or leucovorin or capecitabine).mp. 13362

9 5-fluorouracil.mp. 5739

10 capecitabine.mp. 4752

11 (chemotherap* or radiotherap* or radiation therap* or irradiation or chemorad* or brachytherap*).tw,kw. 112407

12 bevacizumab.mp. 7544

13 (ziv aflibercept or ramucirumab or cetuximab or panitumumab).tw,kw. 3759

14 Irinotecan.mp. 3802

15 Cetuximab.mp. 2603

16 Panitumumab.mp. 802

17 Regorafenib.mp. 664

18 (Trifluridine or tipiracil).mp. 335

19 exp Transanal Endoscopic Surgery/ 36

20 (Proctectom* or surg* or resect* or excision*).tw,kw. 295612

21 pembrolizumab.mp. 3003

22 nivolumab.mp. 2920

23 ((adjuvant or neoadjuvant) adj2 (therap* or treat*)).tw,kw. 21519

24 or/4-23 437906

25 3 and 24 5179

26 exp Rectal Neoplasms/dt, su, rt, th 6

27 25 or 26 5180

28 limit 27 to yr="2000 -Current" 4435

29 conference proceeding.pt. 230423

30 28 not 29 3490

31 limit 30 to trial registry record 1378

32 30 not 31 2112
