# Supplementary material for: Development of a CORe outcome set for clinical trials of RECTal cancer treatment: protocol for the CORRECT initiative
Source: BMJ Open. 2026 Apr 13;16(4):e103072. doi: 10.1136/bmjopen-2025-103072 (PMC13084911; doi:10.1136/bmjopen-2025-103072)
Supplement: Supplementary file 2 [file bmjopen-2025-103072.reviewer_comments.pdf]

PEER REVIEW HISTORY

BMJ Open publishes all reviews undertaken for accepted manuscripts. Reviewers are asked to complete a checklist review form and are provided with free text boxes to elaborate on their assessment. These free text comments are reproduced below.

ARTICLE DETAILS

Title (Provisional)

Development of a CORe outcome set for clinical trials of RECTal cancer treatment: protocol for the CORRECT initiative

Authors

Garfinkle, Richard; George, Manju; Jethwa, Krishan; Johansen, Peter; Lakaszawki, Mary; Nagarajan, Arun; Smart , Neil; Sylla, Patricia; Vuong, Te; Boutros, Marylise; Fergusson, Dean A

VERSION 1 - REVIEW

**Reviewer**	**1**
**Name**	**Leon-Arellano, Miguel**
**Affiliation**	**Hospital Universitario Fundacion Jimenez Diaz**
**Date**	**25-Oct-2025**
**COI**	**None**

This is a clearly written, methodologically rigorous, and well-structured study protocol addressing an important gap in rectal cancer research. The rationale is compelling: heterogeneity in outcome reporting limits synthesis across trials, and the development of a consensus-based core outcome set (COS) will meaningfully enhance comparability and patient-centeredness in future studies.

VERSION 1 - AUTHOR RESPONSE

REVIEWER #1:

Reviewer 1, Comment 1: This is a clearly written, methodologically rigorous, and well-structured study protocol addressing an important gap in rectal cancer research. The rationale is compelling: heterogeneity in outcome reporting limits synthesis across trials, and the development of a consensus-based core outcome set (COS) will meaningfully enhance comparability and patient-centeredness in future studies.

Response to Reviewer 1, Comment 1: Thank you for your comments and review of the manuscript.
